# *LYL1* gene amplification predicts poor survival of patients with uterine corpus endometrial carcinoma: analysis of the Cancer genome atlas data

**DOI:** 10.1186/s12885-018-4429-z

**Published:** 2018-05-02

**Authors:** Se Ik Kim, Ji Won Lee, Nara Lee, Maria Lee, Hee Seung Kim, Hyun Hoon Chung, Jae-Weon Kim, Noh Hyun Park, Yong-Sang Song, Jeong-Sun Seo

**Affiliations:** 1 Department of Obstetrics and Gynecology, Seoul National University College of Medicine, Daehak-Ro, Jongno-Gu, Seoul, Republic of Korea; 2 Gongwu Genomic Medicine Institute (G2MI), Medical Research Center, Seoul National University Bundang Hospital, Seongnam, Republic of Korea; 3 Department of Biomedical Sciences, Seoul National University College of Medicine, Seoul, Republic of Korea; 4 Macrogen Inc., Seoul, Republic of Korea

**Keywords:** Endometrial Neoplasms, The Cancer Genome Atlass, *LYL1*s, Survival analysiss, Gene expression pattern analysis, Gene set enrichment analysis

## Abstract

**Background:**

Somatic amplifications of the *LYL1* gene are relatively common occurrences in patients who develop uterine corpus endometrial carcinoma (UCEC) as opposed to other cancers. This study was undertaken to determine whether such genetic alterations affect survival outcomes of UCEC.

**Methods:**

In 370 patients with UCEC, we analysed clinicopathologic characteristics and corresponding genomic data from The Cancer Genome Atlas database. Patients were stratified according to *LYL1* gene status, grouped as amplification or non-amplification. Heightened levels of cancer-related genes expressed in concert with *LYL1* amplification were similarly investigated through differentially expressed gene and gene set enrichment analyses. Factors associated with survival outcomes were also identified.

**Results:**

Somatic *LYL1* gene amplification was observed in 22 patients (5.9%) with UCEC. Patients displaying amplification (vs. non-amplification) were significantly older at the time of diagnosis and more often were marked by non-endometrioid, high-grade, or advanced disease. In survival analysis, the amplification subset showed poorer progression-free survival (PFS) and overall survival (OS) rates (3-year PFS: 34.4% vs. 79.9%, *P* = 0.031; 5-year OS: 25.1% vs. 84.9%, *P* = 0.014). However, multivariate analyses adjusted for tumor histologic type, grade, and stage did not confirm *LYL1* gene amplification as an independent prognostic factor for either PFS or OS. Nevertheless, MAPK, WNT, and cell cycle pathways were significantly enriched by *LYL1* gene amplification (*P* < 0.001, *P* = 0.002, and *P* = 0.004, respectively).

**Conclusions:**

Despite not being identified as an independent prognostic factor in UCEC, *LYL1* gene amplification is associated with other poor prognostic factors and correlated with upregulation of cancer-related pathways.

**Electronic supplementary material:**

The online version of this article (10.1186/s12885-018-4429-z) contains supplementary material, which is available to authorized users.

## Background

Uterine corpus endometrial cancer (UCEC) imposes a global burden in both developed and developing countries [1]. In the United States, it is the most common gynecologic malignancy, accounting for 61,380 new cases in 2017 [2]. In Korea, the incidence of UCEC is clearly increasing and is estimated to comprise 2.5% (2578) of all new female cancers in 2017 [3, 4].

In 2013, The Cancer Genome Atlas (TCGA) Research Network issued an integrated report of genomic, transcriptomic, and proteomic profiles in 373 patients diagnosed with UCEC [5]. Furthermore, this consortium determined four prognostic categories (good→poor as shown) for classification of UCEC: (1) polymerase ɛ (POLE) ultramutated; (2) microsatellite instability (MSI) hypermutated; (3) low copy number; and (4) high copy number. The high copy number group in particular includes most of the serous and serous-like endometrioid tumors, sharing genomic features with ovarian serous carcinomas. Researchers have since incorporated these molecular criteria into clinical trials designed to gauge postsurgical adjuvant treatment of UCEC (10.1186/ISRCTN11659025).

In keeping with the era of precision medicine, discovery of reliable genetic changes is essential to provide individualized treatment of patients with UCEC [5, 6]. Little-known genes such as *LYL1* may now be identified as novel prognostic indicators or as potential therapeutic targets. The *LYL1* gene is located on the short (p) arm of chromosome 19 at position 13.13, where it encodes a protein implicated in blood vessel maturation and haematopoiesis [7]. As a member of basic helix-loop-helix transcription factor family, the *LYL1* gene is also known to regulate cell proliferation and differentiation [8], and a form of T-cell acute lymphoblastic leukaemia has been linked to a chromosomal aberration of *LYL1* [7].

Curiously, somatic amplifications of the *LYL1* gene frequently accompany UCEC, more so than most other cancers, ranking second among TCGA listings. However, its ramifications in this setting have yet to be fully explored. The current study, entailing TCGA database analysis, was undertaken to determine whether genetic alterations in the *LYL1* gene (such as amplification) may impact survival outcomes in patients with UCEC.

## Methods

### Data acquisition

We downloaded genomic alteration data on patients with UCEC and corresponding clinicopathologic profiles at the Genomics Data Commons (https://portal.gdc.cancer.gov) and cBioPortal for Cancer Genomics (http://www.cbioportal.org) web portals. The Illumina Genome Analyzer served as platform for DNA sequencing (Illumina Inc., San Diego, CA, USA). This study complied with TCGA publication guidelines and policies (http://cancergenome.nih.gov/publications/publicationguidelines). The Institutional Review Board of Seoul National University Hospital ruled that no formal ethics approval was required in this study.

### Study population

In total, 370 patients with UCEC qualified for this study. The clinicopathologic data collected included age, underlying comorbidities, International Federation of Gynecology and Obstetrics (FIGO) stage, tumor histologic type and grade, and treatment of UCEC (ie, surgery, radiation, chemotherapy). Tumor MSI status was also collected. Patients were assigned to *LYL1* gene amplification and non-amplification groups as warranted.

### Bioinformatics analysis

*LYL1* gene status, especially whether it was amplified, was determined through the cBioPortal for Cancer Genomics (http://www.cbioportal.org). Level-3 data of patients with UCEC and raw reads (HTSeq-counts) of differentially expressed gene (DEG) analyses were accessed via FireBrowse (http://firebrowse.org). The Kyoto Encyclopedia of Genes and Genomes (KEGG) pathway analysis of gene expression data [9] was subjected to Gene Set Enrichment Analysis (GSEA) [10]. For visualization of enrichment pathway, the NetworkAnalyst (http://www.networkanalyst.ca) was used [11].

In doing so, the Search Tool for the Retrieval of Interacting Genes/Proteins (STRING) database was applied, achieving confidence scores of 400–1000 [12]. DEGs were identified through open-source software analysis (R package DESeq2; http://www.bioconductor.org) [13].

### Statistical analysis

To compare clinicopathologic features of the two patient subsets, Student’s *t*-test and Mann-Whitney *U*-test were applied to continuous variables, and Pearson’s chi-squared and Fisher’s exact tests for categorical variables.

We defined PFS as the time elapsed between date of initial diagnosis and date of disease progression, whereas overall survival (OS) represented the time interval between date of initial diagnosis and date of cancer-related death or end of study. Survival estimates were generated via Kaplan-Meier method and log-rank test. Cox proportional hazards regression models were engaged to calculate hazard ratios (HRs) and 95% confidence intervals (CIs). For survival analytics, we relied on commercially available software (SPSS v21.0; IBM, Armonk, NY, USA). Open-source programming (R v2.12.1, ISBN 3–900,051–07-0, http://www.R-project.org; R Foundation for Statistical Computing, Vienna, Austria) was used for all other computations. Statistical significance was set at *P* < 0.05.

## Results

### Somatic copy number variations in UCEC

**Fig. 1:**
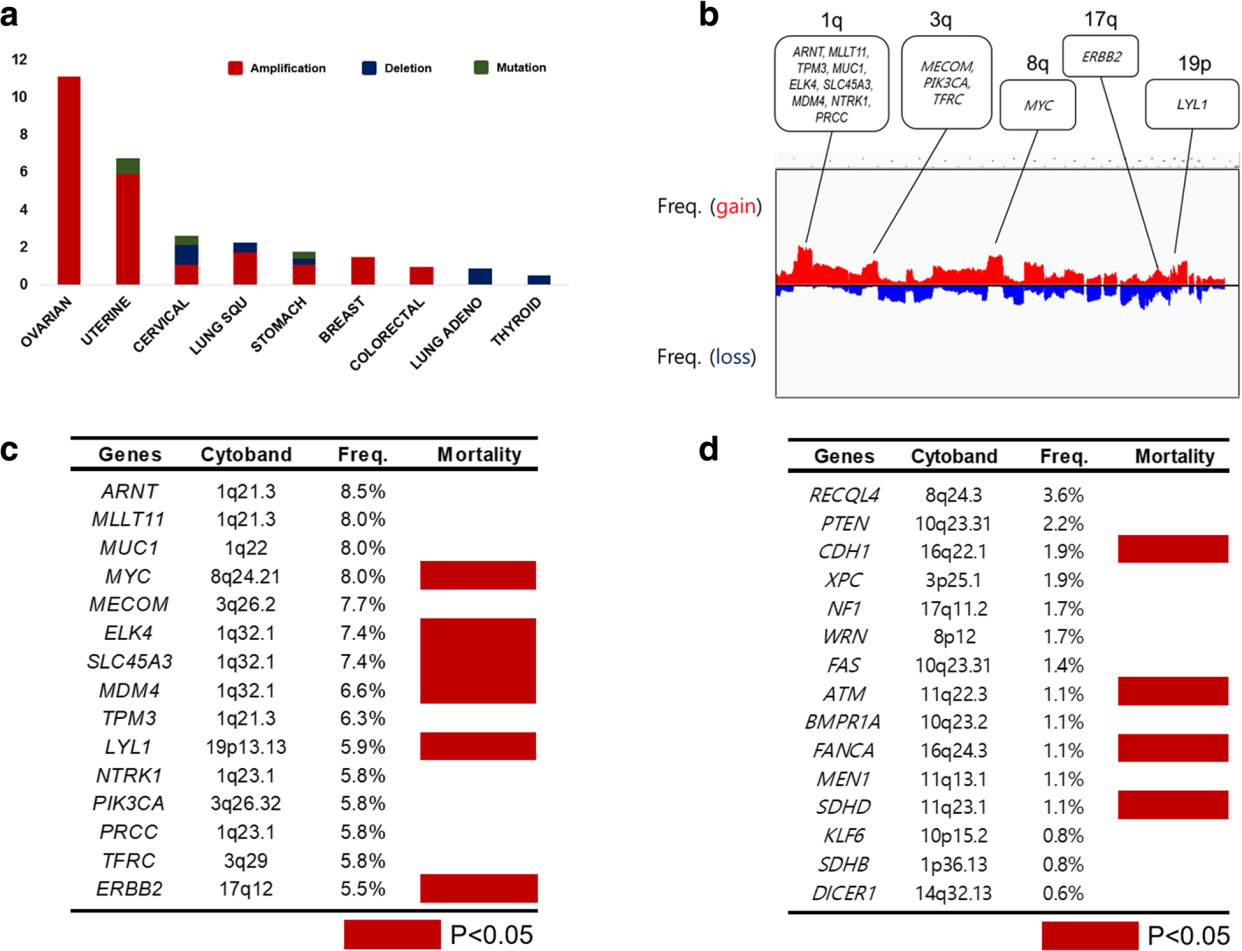
Analysis of gene amplification in various cancer types and uterine corpus endometrial carcinoma: **a** frequencies of copy number variations across chromosomes; **b** frequencies of *LYL1* gene amplification in various cancer types; **c** correlations between amplification frequencies and mortality across top 15 oncogenes; and (**d**) correlations between deletion frequencies and mortality across top 15 tumor suppressor genes in uterine corpus endometrial carcinoma

Frequencies of somatic amplifications involving the *LYL1* gene are depicted according to TCGA classification in Fig. 1a. UCEC ranked second among cancers in terms of *LYL1* gene amplification. In genomic alteration analyses, chromosomes 1q, 3q, 8q, 17q, and 19p were frequently amplified in this patient population (Fig. 1b). The *LYL1* gene of 19p arm was amplified in 5.9% (22/370) of patients with UCEC. Additionally, the *LYL1* gene was one of the 15 mostly amplified oncogenes filtered by gene family in GSEA (Fig. 1c). Meanwhile, the 15 mostly deleted tumor suppressor genes, including *PTEN*, are displayed in Fig. 1d.

### Characteristics of patients with UCEC

Patients’ clinicopathologic characteristics are presented in Table 1. Mean patient age was 63 years. Of the 370 patient participants, 304 (82.2%), 52 (14.1%), and 14 (3.8%) displayed endometrioid, serous, and mixed histologic types of UCEC, respectively. Members of the *LYL1* amplification (vs. non-amplification) group were significantly older at time of diagnosis and more often exhibited biologically aggressive tumors, marked by advanced-stage disease (FIGO stage III-IV; *P* = 0.003), high-grade malignancy (grade 3; *P* < 0.001), and serous histologic type (*P* < 0.001). Proportions of the four TCGA categories of UCEC also showed comparative differences, with 72.7% of amplification group members achieving high copy number rank, versus 12.1% in the non-amplification group (*P* < 0.001). In terms of adjuvant treatment, chemotherapy recipients were more numerous in *LYL1* amplification group than in non-amplification group (50.0% vs 28.4%; *P* = 0.032) (Table 1).

**Table 1:** Clinicopathologic characteristics of patients

Characteristics	All (*n* = 370, %)	*LYL1* amplification (*n* = 22, %)	*LYL1* non-amplification (*n* = 348, %)	*P*
Age, years				< 0.001
Mean ± SD	63.1 ± 11.0	72.7 ± 8.0	62.5 ± 10.9	
Menopause^a^				0.393
Yes	313 (84.6)	0 (0)	27 (7.8)	
No	27 (7.3)	21 (95.5)	292 (83.9)	
Unknown	30 (8.1)	1 (4.5)	29 (8.3)	
Diabetes				0.774
Yes	83 (22.4)	5 (22.7)	78 (22.4)	
No	221 (59.7)	11 (50.0)	210 (60.3)	
Unknown	66 (17.8)	6 (27.3)	60 (17.2)	
Hypertension				0.420
Yes	195 (52.7)	11 (50.0)	184 (52.9)	
No	135 (36.5)	5 (22.7)	130 (37.4)	
Unknown	40 (10.8)	6 (27.3)	34 (9.8)	
Histologic type				< 0.001
Endometrioid	304 (82.2)	6 (27.3)	298 (85.6)	
Serous	52 (14.1)	13 (59.1)	39 (11.2)	
Mixed	14 (3.8)	3 (13.6)	11 (3.2)	
Grade				< 0.001
1	88 (23.8)	0 (0)	88 (25.3)	
2	106 (28.6)	1 (4.5)	105 (30.2)	
3	176 (47.6)	21 (95.5)	155 (44.5)	
FIGO stage				0.003
I	254 (68.6)	9 (40.9)	245 (70.4)	
II	24 (6.5)	2 (9.1)	22 (6.3)	
III	72 (19.5)	9 (40.9)	63 (18.1)	
IV	17 (4.6)	2 (9.1)	15 (4.3)	
Unknown	3 (0.8)	0 (0)	3 (0.9)	
TCGA type				
POLE ultra-mutated	17 (4.6)	0 (0)	17 (4.9)	0.612
MSI hyper-mutated	65 (17.6)	0 (0)	65 (18.7)	0.019
Low copy number	90 (24.3)	0 (0)	90 (25.9)	0.006
High copy-number	58 (15.7)	16 (72.7)	42 (12.1)	< 0.001
Indeterminate	140 (37.8)	6 (27.3)	134 (38.5)	0.292
MSI status				< 0.001
Stable	223 (60.2)	22 (100.0)	201 (57.8)	
Low	19 (5.1)	0 (0)	19 (5.5)	
High	125 (33.7)	0 (0)	125 (35.9)	
Indeterminate	3 (0.8)	0 (0)	3 (0.9)	
Adjuvant treatment				
Chemotherapy only	110 (29.7)	11 (50.0)	99 (28.4)	0.032
Radiation only	55 (14.9)	2 (9.1)	53 (15.2)	0.756
Chemotherapy + Radiation	23 (6.2)	1 (4.5)	22 (6.3)	1.000
Hormone therapy	17 (4.6)	0 (0)	17 (4.9)	0.612

*Abbreviations*: *FIGO* International Federation of Gynecology and Obstetrics, *TCGA* The Cancer Genome Atlas; *POLE* polymerase ɛ, *MSI* microsatellite instability, *SD* standard deviation

^a^Menopause was defined as amenorrhea for 6 months or more

### Between-group comparisons of survival outcomes and identification of prognostic factors

**Fig. 2:**
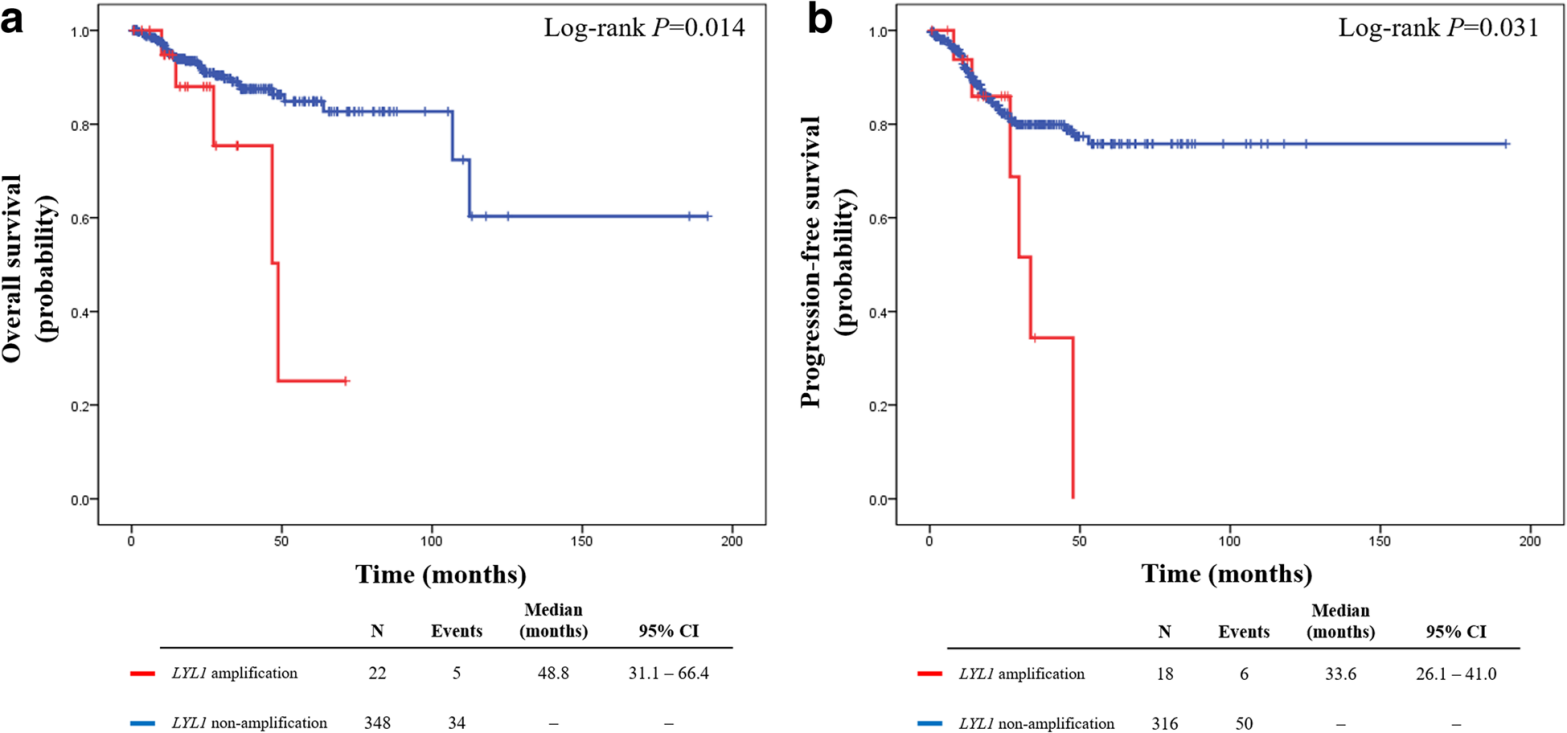
Survival outcomes of patients with uterine corpus endometrial carcinoma, shown by *LYL1* gene status: **a** overall survival and (**b**) progression-free survival

During the observation period (median, 23.9 months; range, 0.5–191.7 months), 5 patients in the amplification group and 34 in the non-amplification group died of their disease. Survival analysis indicated poorer 3-year PFS (34.4% vs. 79.9%; *P* = 0.031) and 5-year OS (25.1% vs. 84.9%; *P* = 0.014) in the amplification (vs. non-amplification) group (Fig. 2).

*LYL1* gene amplification also showed a significant association with poor OS in univariate analysis (*P* = 0.019) (Table 2). However, after adjusting for variables such as histologic type, grade, and FIGO stage, *LYL1* gene status was not confirmed as a significant prognostic factor in OS. Only advanced-stage disease (FIGO stage III-IV) emerged as an independent predictor of poor prognosis (adjusted HR, 3.509; 95% CI, 1.734–7.101; *P* < 0.001). Table 2 also presents factors associated with PFS. In univariate analysis, *LYL1* gene amplification was associated with poor PFS (*P* = 0.037), but its statistical significance was not sustained in multivariate analysis. Advanced-stage disease (FIGO stage III-IV) was identified as an independent poor prognostic factor for PFS (adjusted HR, 3.581; 95% CI, 1.981–6.473; *P* < 0.001).

**Table 2:** Factors associated with survival outcomes in patients with uterine corpus endometrial carcinoma

Characteristics	Overall survival							Progression-free survival						
	N	Univariate analysis			Multivariate analysis			N	Univariate analysis			Multivariate analysis		
		HR	95% CI	*P*	Adjusted HR	95% CI	*P*		HR	95% CI	*P*	Adjusted HR	95% CI	*P*
Age, years														
≥ 63	193	1.456	0.754–2.813	0.263	1.234	0.592–2.570	0.575	157	1.285	0.751–2.197	0.360	1.224	0.676–2.216	0.504
< 63	177	1 (Ref)	−	−	1 (Ref)	−	−	177	1 (Ref)	−	−	1 (Ref)	−	−
Histologic type														
Non-endometrioid	66	2.232	1.146–4.348	0.018	0.887	0.379–2.074	0.782	57	1.987	1.112–3.551	0.020	1.080	0.478–2.442	0.853
Endometrioid	304	1 (Ref)	−	−	1 (Ref)	−	−	277	1 (Ref)	−	−	1 (Ref)	−	−
Grade														
G3	176	3.411	1.661–7.004	0.001	2.016	0.874–4.650	0.100	150	1.539	0.909–2.607	0.109	0.884	0.453–1.724	0.717
G1,2	194	1 (Ref)	−	−	1 (Ref)	−	−	184	1 (Ref)	−	−	1 (Ref)	−	−
FIGO stage														
III-IV	89	4.703	2.474–8.943	< 0.001	3.509	1.734–7.101	< 0.001	72	3.704	2.176–6.307	< 0.001	3.581	1.981–6.473	< 0.001
I-II	278	1 (Ref)	−	−	1 (Ref)	−	−	259	1 (Ref)	−	−	1 (Ref)	−	−
*LYL1* amplification														
Yes	22	3.096	1.201–7.982	0.019	1.581	0.541–4.620	0.402	18	2.469	1.055–5.780	0.037	1.652	0.633–4.317	0.305
No	348	1 (Ref)	−	−	1 (Ref)	−	−	316	1 (Ref)	−	−	1 (Ref)	−	−

*Abbreviations*: *HR* hazard ratio, *CI* confidence interval, *FIGO* International Federation of Gynecology and Obstetrics

We also stratified patients by tumor histologic type for subgroup analysis. In those with endometrioid cancers (*n* = 304), neither PFS (*P* = 0.070) nor OS (*P* = 0.323) differed significantly by *LYL1* gene status (amplification vs. non-amplification). However, results of multivariate analysis showed a trend towards worse PFS in the patients with *LYL1* gene amplification (adjusted HR, 4.093; 95% CI, 0.926–18.012; *P* = 0.063) (Table 3).

**Table 3:** Factors associated with survival outcomes in patients with endometrioid histologic type of uterine corpus endometrial carcinoma

Characteristics	Overall survival						Progression-free survival					
	Univariate analysis			Multivariate analysis			Univariate analysis			Multivariate analysis		
	HR	95% CI	*P*	Adjusted HR	95% CI	*P*	HR	95% CI	*P*	Adjusted HR	95% CI	*P*
Age, years												
≥ 63	1.712	0.773–3.790	0.185	2.044	0.887–4.710	0.093	1.617	0.859–3.045	0.137	1.853	0.955–3.595	0.068
< 63	1 (Ref)	−	−	1 (Ref)	−	−	1 (Ref)	−	−	1 (Ref)	−	−
Grade												
G3	3.041	1.379–6.704	0.006	1.817	0.768–4.302	0.174	1.322	0.702–2.489	0.388	0.889	0.452–1.746	0.733
G1,2	1 (Ref)	−	−	1 (Ref)	−	−	1 (Ref)	−	−	1 (Ref)	−	−
FIGO stage												
III-IV	4.780	2.175–10.506	< 0.001	4.400	1.870–10.354	0.001	3.402	1.762–6.567	< 0.001	4.049	2.013–8.146	< 0.001
I-II	1 (Ref)	−	−	1 (Ref)	−	−	1 (Ref)	−	−	1 (Ref)	−	−
*LYL1* status												
Amplification	2.650	0.355–19.775	0.342	2.823	0.354–22.531	0.327	3.443	0.826–14.347	0.089	4.093	0.926–18.102	0.063
Non-amplification	1 (Ref)	−	−	1 (Ref)	−	−	1 (Ref)	−	−	1 (Ref)	−	−

*Abbreviations*: *HR* hazard ratio, *CI* confidence interval, *FIGO* International Federation of Gynecology and Obstetrics

### DEGs in LYL1 amplified tumors

**Fig. 3:**
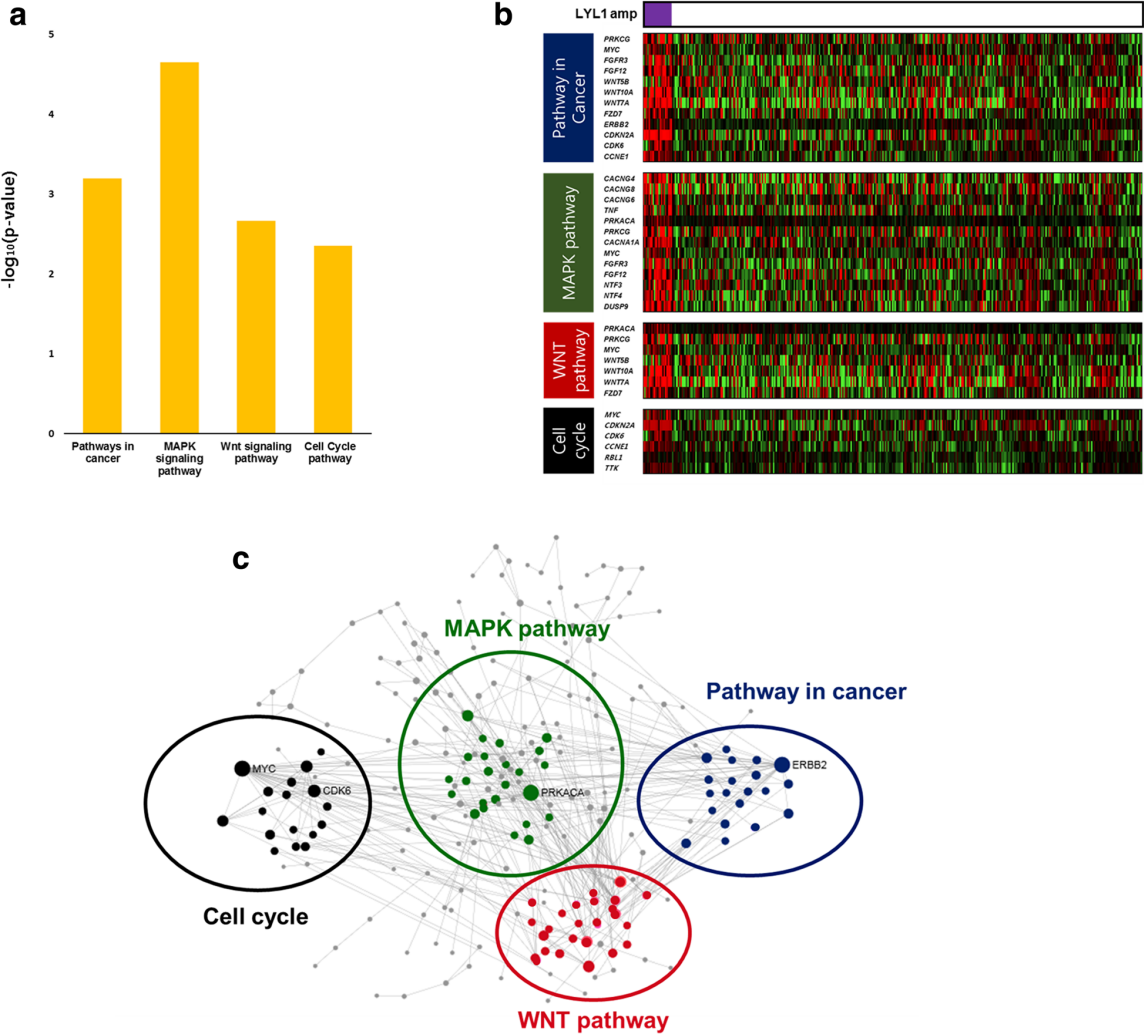
Enrichment analysis of differentially expressed genes (DEGs), shown by *LYL1* gene status: **a** significantly enriched pathway analysis in upregulated 993 DEGs; **b** expression levels of enriched DEGs across *LYL1* amplification; and (**c**) genes in significant gene networks bearing simplified KEGG pathway annotations and grouped process-wise by commonest term prevailing in network (node size defined by degree of interaction)

We performed GSEA pathway analysis of 993 genes showing increased levels of expression in conjunction with *LYL1* amplification. Consequently, we found significant upregulation of MAPK (*P* < 0.001), WNT (*P* = 0.002), cell cycle (*P* = 0.004), and cancer-related (*P* < 0.001) pathways (Fig. 3a, b). Of 993 DEGs, 384 cancer-related genes filtered via STRING database were enriched through these pathways. *MYC, CDK6, PRKACA*, and *ERBB2* genes were found to frequently interact with other cancer-related genes (Fig. 3c).

We also conducted GSEA according to histologic types and TCGA classes (Additional file 1: Figure S1). Among the four TCGA classes, only the high copy number group showed *LYL1* amplifications, and cell proliferation pathway was significantly enriched in this group. Compared to endometrioid type, cancer-related and cell proliferation pathways and genes were more commonly enriched in serous type (Additional file 2: Figure S2).

## Discussion

In the present study, we used TCGA database analysis to determine the potential impact of *LYL1* gene amplification on survival outcomes in patients with UCEC. Although patients displaying *LYL1* gene amplification showed poorer PFS and OS compared to those with non-amplification, multi-variate analyses failed to prove it as an independent prognostic factor.

A number of studies have been similarly conducted to date to identify novel biomarkers for patient survival in various types of cancer. In particular, the prognostic impact made by altered expression levels of *L1CAM* and *MYC*, both homeobox gene family members*,* has been researched through TCGA database analysis [14–16]. The *LYL1* gene, a basic helix-loop-helix transcription factor and a known oncogene in human and mouse cancers, is linked to many cancer-related properties, such as angiogenesis [17–19]. Through genetic and epigenetic modulations, the *LYL1* gene acts to regulate cell proliferation and differentiation [8]. Both in vivo and in vitro experiments have also demonstrated its interactions with various oncogenes, such as *MYC, TAL1, TAL2,* and *LMO2* [20, 21].

Through our TCGA data analysis of *LYL1* gene amplification in patients with UCEC, we discovered that overexpressed cancer-related genes are enriched by MAPK, WNT, and cell cycle pathways in such patients. Specifically, *MYC*, *CDK6*, *PRKACA,* and *ERBB2*, all well-known oncogenes and cancer markers, were overexpressed in conjunction with *LYL1* gene amplification. Both *MYC* and *ERBB2* have likewise shown associations with uterine cancers in earlier studies [22–26]. Additionally, expression of *PRKACA* was positively correlated with *LYL1* amplification (Pearson’s coefficient (r), 0.442).

Unfortunately, only advanced-stage disease emerged as a significant marker of poor prognosis in multivariate analyses. *LYL1* gene amplification was not identified as an independent prognostic factor. However, most of our cohort had early-stage disease (FIGO stages I and II: 68.6% and 6.5%, respectively). According to Surveillance, Epidemiology, and End Results data of the National Cancer Institute, the 5-year survival rate for UCEC with distant metastasis is a dismal 16.2%, compared with 95.3% for disease confined to primary sites [27]. It is thus apparent that the stage of UCEC impacts survival outcomes dramatically, hindering analysis of amplification effects in the current study population.

The current study has several acknowledged limitations, the first being that associations between the *LYL1* gene and other genes or genetic mechanisms were not validated, and the proteins expressed were not measured. Such proteogenomic studies would perhaps underscore the effects of these genetic alterations and the accuracy and completeness of genomic profiling. In addition, further efforts to identify the genetic and epigenetic regulatory mechanisms of the *LYL1* gene and an evaluation of its efficacy as a prognostic indicator and therapeutic target are warranted. In UCEC cell lines, the *LYL1* gene could be overexpressed or inhibited by siRNA, determining subsequent flux in cell differentiation, proliferation, or death. A *LYL1* gene knock-out patient-derived xenograft animal model is one possible investigative approach. Another limitation was the sample size of the *LYL1* gene amplification group (*n* = 22), which was too small for reasonable statistical inferences. Despite these drawbacks, we were able to explore the prognostic potential of the novel *LYL1* gene in the setting of UCEC using both TCGA and clinicopathologic data. *LYL1* gene amplification and its association with expression levels of other genes were demonstrated as well.

## Conclusions

In conclusion, *LYL1* gene amplification is not identified as an independent prognostic factor in UCEC. However, we discovered that cancer-related pathways, such as MAPK, WNT, and cell cycle pathways are upregulated in patients with *LYL1* amplification. Correlations between *LYL1* amplification and increased expression levels of cancer-related genes (*MYC*, *CDK*6, *PRKACA,* and *ERBB2*) are also observed. Its potential for prognostic indicator and therapeutic targeting may be implied based on overexpression of such affiliated oncogenes. Additional multi-omics and genome-wide data studies are warranted.

## Additional files

Additional file 1: **Figure S1.** Gene set enrichment analysis according to histologic types and TCGA classes. (PNG 144 kb)

Additional file 2: **Figure S2.** Enriched genes of cancer-related and cell proliferation pathways according to the two histologic types; serous and endometrioid. (PNG 486 kb)

## Abbreviations

CI: Confidence interval
DEG: Differentially expressed gene
HR: Hazard ratio
KEGG: The kyoto encyclopedia of genes and genomes GSEA Gene set enrichment analysis
MSI: Microsatellite instability FIGO International Federation of Gynecology and Obstetrics
OS: Overall survival
PFS: Progression-free survival
POLE: Polymerase ɛ
STRING: The search tool for the retrieval of interacting genes/proteins
TCGA: The cancer genome atlas
UCEC: Uterine corpus endometrial cancer
