# Supplementary figures and images for: LYL1 gene amplification predicts poor survival of patients with uterine corpus endometrial carcinoma: analysis of the Cancer genome atlas data
Source: BMC Cancer. 2018 May 2;18:494. doi: 10.1186/s12885-018-4429-z (PMC5930686; doi:10.1186/s12885-018-4429-z)

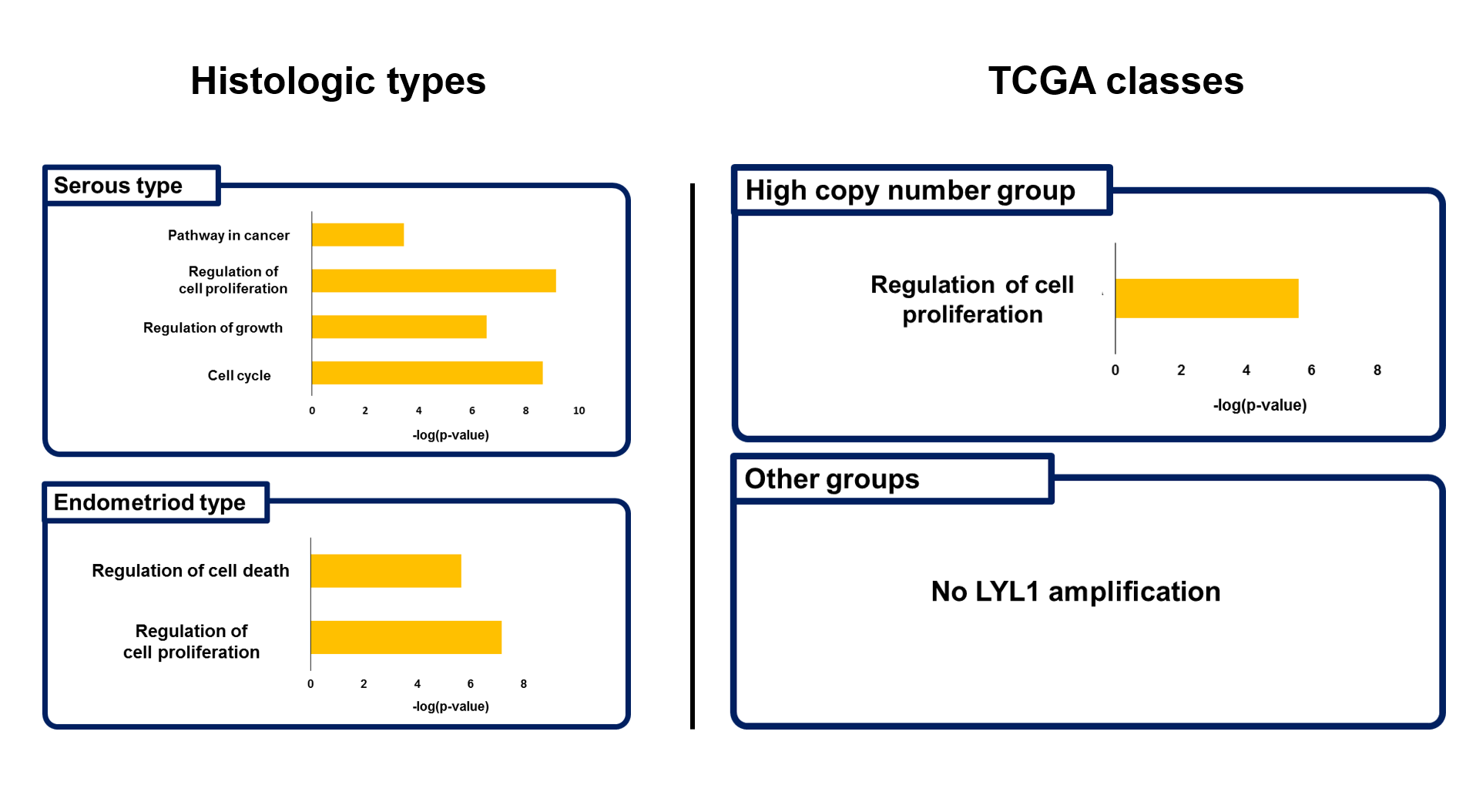

Supplement: Supplementary file 1 — Figure S1. Gene set enrichment analysis according to histologic types and TCGA classes. (PNG 144 kb) [file 12885_2018_4429_MOESM1_ESM.png]

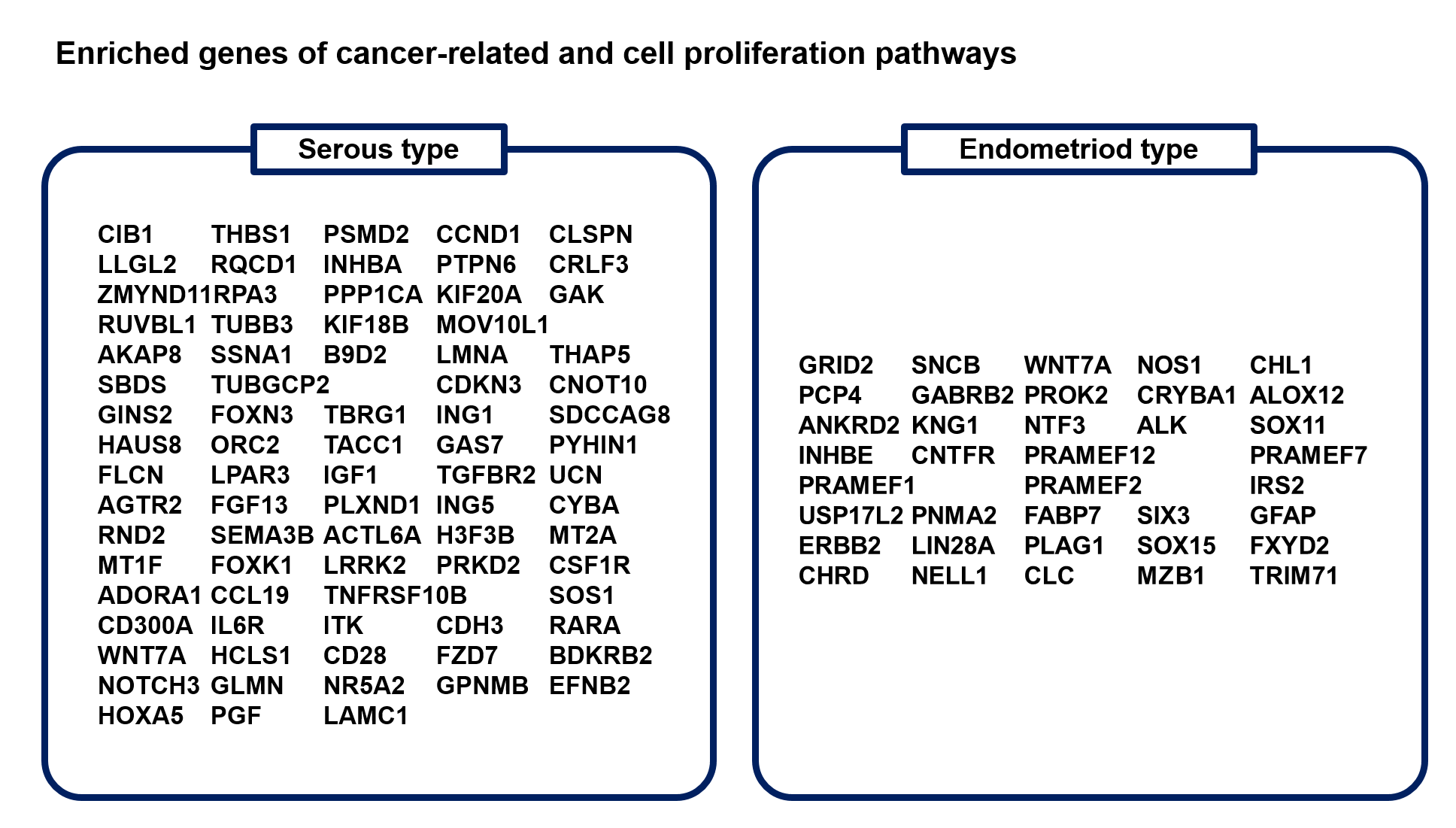

Supplement: Supplementary file 2 — Figure S2. Enriched genes of cancer-related and cell proliferation pathways according to the two histologic types; serous and endometrioid. (PNG 486 kb) [file 12885_2018_4429_MOESM2_ESM.png]
